# Acclimatization of photosynthetic apparatus and antioxidant metabolism to excess soil cadmium in *Buddleja* spp.

**DOI:** 10.1038/s41598-020-78593-8

**Published:** 2020-12-08

**Authors:** Weichang Gong, Bruce L. Dunn, Yaqing Chen, Yunmei Shen

**Affiliations:** 1 College of Life Science and Technology, Honghe University, Mengzi, 661199 Yunnan China; 2 Department of Horticulture and Landscape Architecture, Oklahoma State University, Stillwater, OK USA; 3 College of Teacher Education, Honghe University, Mengzi, 661199 Yunnan China

**Keywords:** Abiotic, Plant physiology

## Abstract

Heavy metal (HM) pollutants can cause serious phytotoxicity or oxidative stress in plants. *Buddleja* L., commonly known as “butterfly bushes”, are frequently found growing on HM-contaminated land. However, to date, few studies have focused on the physiological and biochemical responses of *Buddleja* species to HM stress. In this study, potted seedlings of *B*. *asiatica* Lour. and *B*. *macrostachya* Wall. ex Benth. were subjected to various cadmium (Cd) concentrations (0, 25, 50, 100, and 200 mg kg^−1^) for 90 days. Both studied *Buddleja* species showed restricted Cd translocation capacity. Exposure to Cd, non-significant differences (*p* > 0.05) were observed, including quantum yield of photosystem II (PSII), effective quantum yield of PSII, photochemical quenching and non-photochemical quenching in both species between all studied Cd concentrations. Moreover, levels of cellular reactive oxygen species (ROS) significantly declined (*p* < 0.05) with low malondialdehyde concentrations. In *B. asiatica*, high superoxide dismutase and significantly enhanced (*p* < 0.05) peroxidase (POD) activity contributed greatly to the detoxification of excess ROS, while markedly enhanced POD activity was observed in *B. macrostachya*. Additionally, *B. macrostachya* showed higher membership function values than did *B. asiatica*. These results suggested that both *Buddleja* species exhibited high Cd resistance and acclimatization.

## Introduction

Heavy metal (HM) pollution is a serious environmental problem, and the area of land contaminated with HM is growing rapidly and endangering animals and plants. Cadmium (Cd) is an extremely dangerous and widespread pollutant^1^. Furthermore, not only is Cd, in contrast to several other HMs, a biologically non-essential nutrient^1,2^, but the use of Cd is also increasing due to the presence of Cd in rocks mined for widely applied phosphate fertilizers and because of other human agricultural behavior^3^. In plants, Cd-induced phytotoxicity or plant oxidative stress is a complex phenomenon, involving plant morphological, physiological, and biochemical responses^4^. Physical symptoms of Cd-induced phytotoxicity include leaf chlorosis (such as reduced chloroplast organization, or impaired photosynthetic pigment or enzyme function) or withering, growth retardation (e.g., altered root–shoot ratios), reduction of photosynthetic activity, and lipid peroxidation^5–11^.

Under HM stress, plants generally overproduce reactive oxygen species (ROS), which disrupts the intracellular balance and raises the degree of lipid peroxidation, incurring severe redox reactions^12,13^. Cd is known to both induce the generation of excess ROS^10,14^, and to inhibit enzyme activity by masking catalytically active groups through interaction with their ligands^15^. It therefore not only causes damage to the enzymatic systems of cells^11,16,17^, but simultaneously causes damage to the leaf photosynthetic apparatus (e.g., photosynthetic Calvin-Benson cycle), and impairs the metabolism of phosphorus (P) and nitrogen (N), leading to declines in photosynthesis^8,10,12^. To overcome Cd-induced toxicity, plants have developed diverse defensive mechanisms to lessen or detoxify oxidative stress, such as enzymatic and non-enzymatic antioxidants, and osmoprotectants^1,16–21^. In certain tolerant plants, or hyperaccumulators, enzymatic antioxidants, including superoxide dismutase (SOD), peroxidase (POD) and catalase (CAT) can efficiently break down ROS and can thereby maintain intracellular ROS at a moderate level^8,11,16,17^. Production of antioxidative enzymes is an important part of the plant defense system in the protection against various environmental stresses. Because of the high efficiency of these mechanisms in counteracting oxidative stress, they are considered to be a key mechanism in evaluating the tolerance and fitness of plant species^11,17^.

Photosystem II (PSII) is known to be more sensitive to HM stress than photosystem I (PSI)^22^. Chlorophyll a fluorescence of PSII has often been used as a tool to determine the effects of abiotic stress on the photosynthetic apparatus^22^. Moreover, the evaluation of chlorophyll a fluorescence can also provide indirect information about plant physiological responses and growth performance under any growth conditions or abiotic stresses^11,22,23^. Chlorophyll fluorescence represents the energy that is re-emitted from chlorophyll molecules that return from an excited state to a non-excited state. Chlorophyll molecules in their excited state are able to dissipate absorbed light either through photochemical processes, especially photosynthesis^22,24^, or by emitting fluorescence. Cd-induced stresses may not only cause seriously damage to the photosynthetic apparatus, reducing the photosynthetic capacity, but may also reduce plant fitness^1,11,22–24^.

*Buddleja* L. (Scrophulariaceae) species, commonly known as “butterfly bushes”, are widely distributed throughout Asia, Africa, and America^25^. *Buddleja* plants are commonly found along roadsides or abandoned land, but also grow in HM-contaminated land, and several species are known to have the capacity to accumulate large amounts of heavy metals. *Buddleja asiatica* Lour., *B. paniculata* Wall., *B. scordioides* Kunth and *B. tomentella* Standl. have shown great Pb tolerance, uptake and accumulation potential in various situations, including at mine tailing disposal sites^26^. Qi et al.^27^ found that *Buddleja davidii* L. can grow naturally at Xikuangshan mining area, where the soil is contaminated with antimony (Sb). However, although several *Buddleja* species are known to be able to tolerate HM-induced stress, the physiological and biochemical response mechanisms, in particular regarding Cd stress, are not well understood.

The toxic effects of Cd on plant growth and metabolism differ among plant species. In this study, we evaluated the physiological and biochemical responses of *B*. *asiatica* Lour. and *B*. *macrostachya* Wall. ex Benth. to increasing soil Cd concentrations. The objective of this study was (1) to determine the capacity of two *Buddleja* species to bioaccumulate Cd; (2) to investigate physiological and biochemical mechanisms to prevent Cd toxicity in these two species; and (3) to evaluate the potential of these two species to acclimatize to Cd-contaminated land.

## Results

### Cd uptake, transfer, and bioaccumulation

The Cd concentrations, the calculated translocation and bioconcentration factors in *B. asiatica* and *B. macrostachya* are listed in Table 1. After being cultivated for 90 days, the amount of Cd that had accumulated in both *Buddleja* species was slightly higher when the plants had been grown on increased soil Cd concentrations. In this study, large amounts of Cd were taken up by the roots, 18.17 ± 0.89–75.94 ± 9.71 and 8.54 ± 0.03–184.31 ± 0.27 mg kg^−1^ DW in *B. asiatica* and *B. macrostachya*, respectively (Table 1). In *B. asiatica*, the roots accumulated 75.94 ± 9.71 mg kg^−1^ DW following the 200 mg kg^−1^ Cd treatments, but only 26.15 ± 1.52 and 28.23 ± 1.18 mg kg^−1^ DW Cd had accumulated in the stems and leaves, respectively (Table 1). Following the 200 mg kg^−1^ Cd treatment, *B. macrostachya* had accumulated 184.31 ± 0.27, 32.11 ± 1.89, and 5.43 ± 0.03 mg Cd kg^−1^ DW in the roots, stems, and leaves, respectively (Table 1). The amounts of accumulated Cd in plant tissues (including root, stem and leaf) markedly increased with increasing soil Cd concentrations (Table 1). In both study species, the maximum concentrations of Cd in leaves were found following the 50 mg kg^−1^ Cd treatment, with 29.25 ± 2.34 and 6.54 ± 0.13 mg Cd kg^−1^ DW in *B. asiatica* and *B. macrostachya*, respectively (Table 1).

**Table 1:** Characteristics of cadmium uptake, transfer, and accumulation (mean ± S.E.; n = 3) of *B. asiatica* and *B. macrostachya* under different Cd treatments.

Treatment (mg Cd kg^−1^ dry weight soil)	Content (mg Cd kg^−1^ DW; mean ± SE)			BCF		TF
	Root	Stem	Leaf	Root	Shoot	
***Buddleja asiatica***						
0	18.17 ± 0.89a	16.46 ± 3.37a	17.81 ± 2.97a	–	–	1.91 ± 0.25a
25	34.52 ± 5.31a	16.92 ± 0.08a	22.54 ± 1.01a	1.38 ± 0.21a	1.58 ± 0.04a	1.20 ± 0.18ab
50	37.29 ± 2.63a	22.65 ± 0.36a	29.25 ± 2.34b	0.75 ± 0.10b	1.04 ± 0.05b	1.41 ± 0.12ab
100	75.00 ± 9.90b	22.38 ± 2.81a	27.38 ± 0.65b	0.73 ± 0.17b	0.50 ± 0.03 cd	0.70 ± 0.14bc
200	75.94 ± 9.71b	26.15 ± 1.52a	28.23 ± 1.18b	0.38 ± 0.05b	0.27 ± 0.01d	0.74 ± 0.08c
***Buddleja macrostachya***						
0	8.54 ± 0.03a	3.63 ± 0.05a	3.19 ± 0.06a	–	–	0.80 ± 0.01a
25	146.48 ± 0.29b	5.70 ± 0.22a	4.16 ± 0.23b	5.86 ± 0.01a	0.39 ± 0.02a	0.07 ± 0.00b
50	140.35 ± 1.33b	4.43 ± 0.20a	6.54 ± 0.13e	2.81 ± 0.03b	0.22 ± 0.01b	0.08 ± 0.00b
100	173.99 ± 4.36c	6.80 ± 0.38a	4.99 ± 0.20c	1.74 ± 0.04c	0.12 ± 0.00d	0.07 ± 0.00b
200	184.31 ± 0.27d	32.11 ± 1.89b	5.43 ± 0.03d	0.92 ± 0.00d	0.19 ± 0.01bc	0.20 ± 0.01c

Different letters indicate different significant differences (One-way ANOVA; *p* < 0.05).

To evaluate the Cd accumulation capacity of the two *Buddleja* species, the bioconcentration factor (BCF) and translocation factor (TF) were calculated (Table 1). At soil treatments of 25–200 mg Cd kg^−1^, both *B. asiatica* and *B. macrostachya* showed low BCFs, ranging from 0.12 ± 0.00–5.86 ± 0.01 (Table 1). Moreover, the roots of *B. macrostachya* had greater BCFs than those of *B. asiatica*, while, *B. asiatica* shoots presented greater BCFs than those of *B. macrostachya* at the same Cd concentrations (Table 1). In *B. asiatica*, the shoots had relatively greater BCFs than their roots; in contrast, *B. macrostachya* roots had higher BCFs than did the shoots (Table 1).

Compared with *B. macrostachya, B. asiatica* plants exhibited high TF at the same Cd concentrations (Table 1). At soil Cd concentrations of 50 mg Cd kg^−1^ and higher, neither of our study plant species were able to uptake, accumulate, or translocate Cd to the same extent as hyperaccumulators with low TFs (< 1.0). However, at soil Cd concentrations of 50 mg kg^−1^ and less, the TFs values in *B. asiatica* plants were greater than one, ranging from 1.91 ± 025–1.20 ± 0.18 (Table 1). These, however, dramatically declined (*p* > 0.05) with increasing soil Cd concentrations.

### Photosynthetic activity

**Figure 1:**
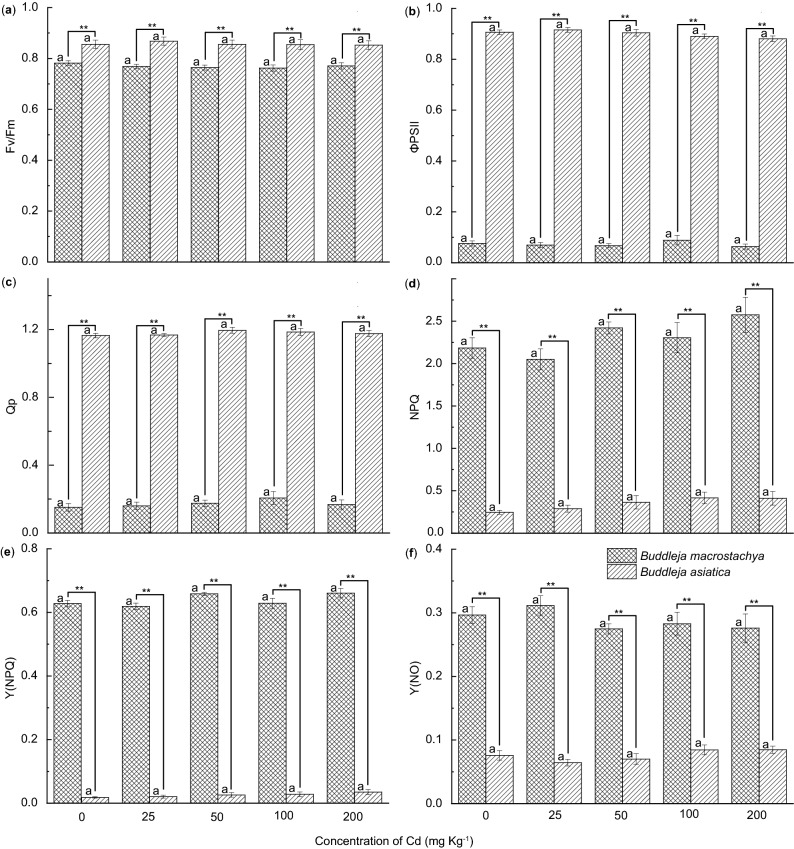
The effects of Cd treatments on (**a**) Fv/Fm, (**b**) ΦPSII, (**c**) Qp, (**d**) NPQ, (**e**) Y(NPQ), and (**f**) Y(NO) in the leaves of *B. asiatica* and *B. macrostachya*. Values are represented as the mean ± S.E. (n = 5). Asterisks indicate significant differences between different species at the same Cd level according to LSD-tests (**, *p* < 0.01). (**a**–**f**) were separately created by using ORIGIN (Version 2019b, OriginLab Corporation, USA) and then adjusted and assembled using Adobe Illustrator CS4 software (Adobe Systems, San Jose, CA). (Fv/Fm): the quantum yield of PSII, ΦPSII: the effective quantum yield of PSII, Qp: photochemical quenching, NPQ: non-photochemical quenching, Y(NPQ): the quantum of regulated energy dissipation, Y(NO): the quantum yield of non-regulated energy dissipation.

To assess the effect of soil Cd on *Buddleja* photosynthesis, leaf chlorophyll *a* fluorescence was determined in our two *Buddleja* study species, *B*. *asiatica* and *B. macrostachya* (Fig. 1). Both species showed similar response patterns to different Cd concentrations within a species, while there were divergent patterns between species (Fig. 1). Both *B*. *asiatica* and *B. macrostachya* showed great quantum yield of PSII (Fv/Fm), 0.86 ± 0.02–0.87 ± 0.02 and 0.77 ± 0.01–0.78 ± 0.01, respectively (Fig. 1a). Following exposure to soil Cd, though *B. asiatica* demonstrated a larger effective quantum yield of PSII (ΦPSII) and greater photochemical quenching (Qp) than did *B. macrostachya* with significant differences (*p* < 0.05), the two species showed similar response patterns to increasing Cd concentrations (Fig. 1b,c). Furthermore, there were no significant differences (*p* > 0.05) between treatments with different soil Cd concentrations at a species level.

Following exposure to Cd, *B. asiatica* plants showed lower non-photochemical quenching (NPQ), quantum of regulated energy dissipation [Y(NPQ)], and quantum yield of non-regulated energy dissipation [Y(NO)] than that in *B. macrostachya* (Fig. 1d–f). Furthermore, there were significant differences (*p* < 0.05) in NPQ, Y(NPQ) and Y(NO) between the two studied *Buddleja* species at all investigated soil Cd concentrations. However, Cd exposure did not increase their NPQ, Y(NPQ) and Y(NO) levels, with only nonsignificant differences (*p* > 0.05).

### Membrane lipid peroxidation

**Figure 2:**
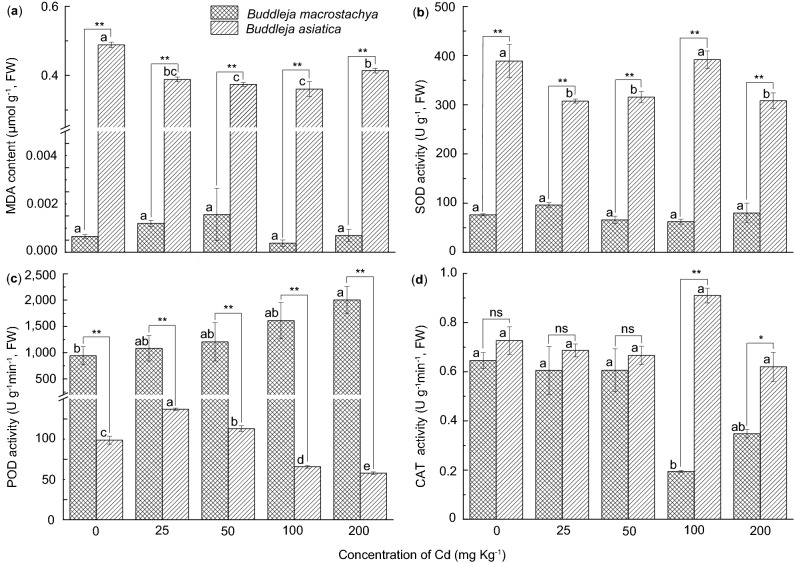
The effects of Cd treatments on (**a**) MDA content, (**b**) SOD activity, (**c**) POD activity, and (**d**) CAT activity in the leaves of *B. asiatica* and *B. macrostachya*. Values are represented as the mean ± S.E. (n = 3). Different letters indicate significant differences between different Cd treatments according to LSD-tests (*p* < 0.05); Asterisks indicate significant differences between different species at the same Cd level according to LSD-tests (ns, nonsignificant; *, *p* < 0.05; **, *p* < 0.01). (**a**–**d**) were separately created by using ORIGIN (Version 2019b, OriginLab Corporation, USA) and then adjusted and assembled using Adobe Illustrator CS4 software (Adobe Systems, San Jose, CA). Abbreviations: MDA: malondialdehyde, SOD: superoxide dismutase, POD: peroxidase, CAT: catalase.

After exposure to Cd for 90 days, seedlings of both *Buddleja* species survived and appeared to be growing normally. To evaluate the membrane lipid peroxidation, we measured the malondialdehyde (MDA) content in the leaves (Fig. 2a). There were significant differences (*p* < 0.05) in the leaf MDA concentrations between *B. asiatica* and *B. macrostachya* at all concentrations of Cd studied (Fig. 2a). In our study, *B. macrostachya* leaves showed consistently lower MDA concentrations (always lower than 0.002 μmol g^−1^) than that did *B. asiatica* leaves, which had MDA concentrations ranging from 0.36 ± 0.02 to 0.49 ± 0.01 μmol g^−1^ (Fig. 2a).

The two *Buddleja* species showed different patterns of MDA response to increasing soil Cd concentrations (Fig. 2a). At Cd concentrations up to 100 mg kg^−1^, the concentration of MDA in *B. asiatica* leaves significantly decreased (*p* < 0.05) with increasing soil Cd concentrations (Fig. 2a). However, leaf MDA concentrations in these plants markedly increased up to a relatively high levels (200 mg Cd kg^−1^) of Cd (Fig. 2a). However, the leaf MDA concentrations in *B. macrostachya* leaves remained at a low level with no significant differences following increasing soil Cd concentrations (*p* > 0.05; Fig. 2a).

### Antioxidant enzyme activities

To evaluate the antioxidant enzyme defense system in our two *Buddleja* study species in response to different soil Cd concentrations, we measured the activities of several enzymatic antioxidants in the leaves (Fig. 2b–d). Following exposure to Cd, *B. asiatica* and *B. macrostachya* showed different response patterns in SOD and POD activities (Fig. 2b,c). Compared with *B. macrostachya* (62.39 ± 5.05 to 96.17 ± 4.71 U g^−1^), *B. asiatica* presented high SOD activity (307.65 ± 3.94–391.69 ± 17.92 U g^−1^) at all investigated soil Cd concentrations (Fig. 2b). Moreover, SOD activity significantly declined (*p* < 0.05) with increasing soil Cd concentrations in *B. asiatica* species, besides the markedly increase at 100 mg Cd kg^−1^ (Fig. 2b). The highest SOD activity occurred following the 100 mg Cd kg^−1^ and control treatments, 391.69 ± 17.92 and 388.83 ± 33.78 U g^−1^, respectively (Fig. 2b). In contrast, *B*. *macrostachya *showed much lower SOD activity (*p* < 0.05) compared to *B. asiatica* at all Cd concentrations. Furthermore, there were no significant differences (*p* > 0.05) between any of the investigated Cd treatments with increasing soil Cd concentrations (Fig. 2b). SOD activity in *B*. *macrostachya *peaked following the 25 mg Cd kg^−1^ treatment, with a value of 96.17 ± 4.71 U g^−1^ (Fig. 2b).

In contrast, POD activity responded very differently to increasing Cd concentrations than did SOD activity in both *B. asiatica* and *B. macrostachya* (Fig. 2b,c). After exposure to soil Cd, *B*. *macrostachya* showed greater POD activity than *B. asiatica* at all Cd concentrations, with values ranging from 943.75 ± 168.14–2,002.92 ± 253.57 U g^−1^ min^−1^ and 57.87 ± 1.63–137.24 ± 1.37 U g^−1^ min^−1^, respectively (Fig. 2c). Up to 200 mg Cd kg^−1^, POD activity in *B. macrostachya* showed a gradual increase with increasing soil Cd concentrations, but the differences between all Cd treatments were nonsignificant (*p* > 0.05). In *B. asiatica*, the POD activity increased at low Cd concentrations (25 mg Cd kg^−1^) with significantly (*p* < 0.05) differences compared to the control (Fig. 2c). However, POD activity declined significantly (*p* < 0.05) following exposure to Cd concentrations above 25 mg kg^−1^ (Fig. 2c).

After exposure to low concentrations of Cd, both *B. asiatica* and *B. macrostachya* presented low catalase (CAT) activities with a similar response pattern (Fig. 2d), and at Cd concentrations up to 50 mg kg^−1^, CAT activity was similar in both between and within species with nonsignificant differences (*p* < 0.05; Fig. 2d). However, following exposure to high soil Cd concentrations (100 and 200 mg Cd kg^−1^), *B. asiatica* plants showed high CAT activities with nonsignificant differences (*p* < 0.05), while those in *B. macrostachya* markedly decreased (Fig. 2d).

### Membership function

To evaluate the Cd resistance and accumulation characteristics in *B*. *asiatica* and *B*. *macrostachya*, a membership function based on all the investigated physiological and biochemical parameters was calculated (Table 2). Following exposure to increasing soil concentrations of Cd, *B. macrostachya* presented greater comprehensive membership function values (D, 6.82–10.04) than did *B. asiatica* (5.00–7.17) at all studied Cd treatments. Compared to the control, exposure to increasing concentrations of Cd caused the D values in *B. asiatica* to decline, while the D values in *B. macrostachya* were increased markedly (Table 2). Moreover, *B. macrostachya* plants showed higher D value (8.88) than that in *B. asiatica* (5.94) at a species level (Table 2).

**Table 2:** The membership function (D) of *B. asiatica* and *B. macrostachya* under different Cd stresses.

Treatment (mg Cd kg^−1^ dry weight soil)	Membership function value (D)	
	*Buddleja asiatica*	*Buddleja macrostachya*
0	7.16	6.82
25	5.11	8.70
50	5.00	9.39
100	5.87	10.04
200	5.18	8.46
Species level	5.94	8.88

## Discussion

Phytoremediation is considered to be an excellent choice for environmental HM management. Plant species have evolved different mechanisms to cope with HM exposure, either acting as contaminant accumulators or excluders^27^. The HM accumulation capacity of hyperaccumulator and hypertolerant plant species has been widely discussed^5,11,17,23,28^. In our present study, both *Buddleja* species extracted and accumulated large amounts of Cd in the roots, but not in the stems or leaves (Table 1). Moreover, the Cd concentrations in all studied plant tissues markedly increased with increasing soil Cd concentrations. According to the standard of Sun et al.^17^, the Cd concentrations in neither roots nor shoots in either *Buddleja* species reached the criterion to qualify as a Cd-hyperaccumulator. Thus, our results suggest that neither of our studied *Buddleja* species would qualify as Cd hyperaccumulators.

To further evaluate the potential for Cd phytoextraction and translocation, translocation factor (TF) values, which can estimate the efficiency of translocation of HMs from plant roots to shoots have often been used^17,28,29^. In our study, *B. asiatica* presented greater TF values than *B. macrostachya* at all studied Cd concentrations (Table 1). Furthermore, at low soil Cd concentrations (e.g., 25 and 50 mg kg^−1^), *B. asiatica* showed a high TF that was larger than one, but this was not the case in *B. macrostachya* (Table 1). This is consistent with Waranusantigul et al.^26^, in which *B. asiatica* showed high Cd translocation potential with large TF values. In addition, BCF plays a crucial role in evaluating the potential for phytoextraction of a given species^17,23,29^. BCF is a measure of the capacity of plants to extract HMs from the soil and transfer them to plant tissues. Although all our data imply that neither of our *Buddleja* study species meets the criteria to qualify as Cd-hyperaccumulators, they still have great potential in HM-contaminated land as typical woody pioneer plants with great soil Cd hypertolerance.

Cd-induced phytotoxicity or redox reactions often causes the damage to the photosynthetic apparatus^6–8,23^. In angiosperms, the damage to the photosynthetic apparatus causes an increase in the Fo value and a decline in the Fv/Fm ratio^30^. The Fv/Fm ratio, which usually ranges between 0.75 and 0.85, has been used as an efficiency indicator to evaluate abiotic stress on PSII^30–32^. In the present study, although the Fv/Fm ratio declined slightly with increasing soil Cd concentrations, both *B. asiatica* (0.86 ± 0.02 to 0.87 ± 0.02) and *B. macrostachya* (0.77 ± 0.01 to 0.78 ± 0.01) continued to show high Fv/Fm ratios (Fig. 1a). Moreover, no significant differences (*p* > 0.05) were found between different Cd treatments within each species. This suggested that both *B. asiatica* and *B. macrostachya* performed well when grown in artificially Cd polluted soil. Furthermore, this implies that both studied *Buddleja* species showed good Cd resistance and tolerance.

Chlorophyll fluorescence competes for excitation energy with photochemical processes, and it can efficiently reflect the leaf photochemical efficiency and growth adaptability of plant species to environmental stresses^22,24^. ΦPSII and Qp are often used to account for practical photosynthetic performance and photosynthetic activity, respectively^22,32^. Compared to *B. macrostachya*, *B. asiatica* plants exhibited higher ΦPSII and Qp with significant differences (*p* < 0.01) at all Cd treatments (Fig. 1b,c). Moreover, there was a positive correlation between ΦPSII and Qp in both *Buddleja* species (Fig. 1b,c). However, no significant differences (*p* > 0.05) were found between the different Cd treatments within each species. Wu et al.^23^ found that Cd-hypertolerant plants or hyperaccumulators may either show an increase in plant net biomass, or exhibit an improved photosynthetic capacity after exposure to Cd. Thus, all our data suggest that Cd exposure did not reduce either plant photosynthetic activity or performance in either *Buddleja* species. Furthermore, both *B. asiatica* and *B. macrostachya* plants presented great photochemical light energy utilization.

Plant species not only can utilize most of the energy in light for photosynthesis, but also can efficiently dissipate the excessive excitation energy into harmless heat^33,34^. The extent of heat dissipation is closely related to the NPQ capacity^35^. The efficiency of the dissipation of excessive excitation energy into harmless heat is also reflected by Y(NPQ)^33,34^. A high Y(NPQ) indicates that the absorbed light energy is excessive and that the excessive excitation energy is efficiently dissipated into harmless heat^33^. In our study, *B. macrostachya* presented greater NPQ and Y(NPQ) capacities compared to *B. asiatica* plants, with significant differences (*p* < 0.01) between the two species (Fig. 1d,e). Nonsignificant differences (*p* > 0.05) were found between different Cd treatments within each species. This suggested that Cd exposure did not reduce the capacity to dissipate excessive excitation energy in either *Buddleja* species. Moreover, *B. macrostachya* presented a greater photoprotection capacity than did *B. asiatica* species.

However, not all PSII energy dissipation can be regulated through NPQ^33,34^. For instance, the non-regulated Y(NO) is made up of nonphotochemical quenching due to photoinactivation and constitutive thermal dissipation^35^. Generally, the value of Y(NO) has been found to be positively associated with the extent of photodamage^33,34^ In this study, Cd exposure did not significantly (*p* > 0.05) increase the values of Y(NO) in both studied *Buddleja* species under any of the Cd treatments. Compared with *B. asiatica*, *B. macrostachya* plants exhibited high Y(NO) values (as NPQ and Y(NPQ)) (Fig. 1f). This implied that exposure to Cd did not increase the extent of photodamage in either of the *Buddleja* species, and that their protective regulatory mechanisms were efficient. Furthermore, our results suggested that both *B. asiatica* and *B. macrostachya* showed high tolerance and acclimatization to artificial soil Cd stress.

Environmental stress often causes oxidative damage and can lead to the excessive production of ROS harming the cell membrane^13,36^. Both stressed and unstressed plant cells might produce ROS, but HM-tolerant plants generally have a well-developed antioxidant system for the removal of ROS^11,23,36^. Malondialdehyde, a product of lipid peroxidation has been used as a key indicator for the determination of oxidative damage in plants^8,11,23,36^. In this study, leaves from both *Buddleja* study species produced low amounts of MDA following treatment with elevated Cd concentrations (Fig. 2a). This implies that both studied *Buddleja* species can efficiently activate their antioxidant defense systems and quench free radicals. Furthermore, these efficient ROS scavenging mechanisms protect *Buddleja* species from destructive reactions under soil Cd stress.

Plants have evolved diverse mechanisms and various protective antioxidant defense systems to eradicate oxidative stress^16,18,20,21^. Antioxidants’ response against Cd toxicity varies among different plant species and experimental conditions^17,19,23,36,37^. Enzymatic antioxidants are predominantly produced in the sensitive foliage and protect plants from damage by quenching free radicals. Compared with Cd-sensitive plants, Cd-tolerant plants response positively in antioxidant enzyme activities to Cd-induced stress^38^. Superoxide dismutase, which is a first line of defense against ROS, can successfully catalyze the disputation of O_2_^−^ to H_2_O_2_ and O_2_^13,37^. POD and CAT activities can effectively decompose H_2_O_2_ at the intracellular level, and convert it into H_2_O^16,17^. In this study, both two studied *Buddleja* species can efficiently regulate the steady-state level of cellular ROS through different antioxidant mechanisms. After exposure to soil Cd stress, *B. asiatica* maintained high SOD levels at all soil Cd concentrations, while high POD levels were maintained in *B. macrostachya* (Fig. 2b,c). *Buddleja asiatica* markedly reduced the activities of SOD and POD following exposure to Cd, however, POD activity significantly improved (*p* < 0.01) following the 25 and 50 mg Cd kg^−1^ treatments (Fig. 2b,c). In contrast, POD activity in *B. macrostachya* gradually increased following exposure to soil Cd, and a stable low level of SOD activity was maintained throughout (Fig. 2b,c). All our results suggest that both our *Buddleja* study species were equipped with efficient mechanism to manage cellular redox homeostasis at its optimum after exposure to soil Cd stress. These enzymatic antioxidants improved plant tolerance to soil Cd stress, and preserved normal plant growth and metabolism in these species following increasing soil Cd concentration.

In addition, to evaluate overall plant resistance and acclimatization to environmental stresses, the comprehensive membership function value (D) was also calculated^39,40^. The D value is used to identify Cd-resistant plants, where a large D value is positively correlated with high resistance and acclimatization^39,40^. After exposure to soil Cd stress, both *B. asiatica* and *B. macrostachya* exhibited not only different response mechanisms and growth performance, but also excellent acclimatization with high D values (Table 2). Combined with their capacities to accumulate Cd in their tissues, our results suggest that *B. asiatica* and *B. macrostachya* show species-specific responses in their resistance and acclimatization to Cd biotoxicity. Moreover, they have efficient ROS detoxification mechanisms in response to soil Cd stress, and are potentially Cd-hypertolerant plants.

## Conclusion

*Buddleja asiatica* and *B. macrostachya* plants growing in Cd-contaminated soil presented high tolerance to Cd stress with limited Cd accumulation capacity. In neither *Buddleja* species did Cd exposure affect their photosynthetic activity or photoprotection capacity. On the contrary, levels of ROS significantly declined, the plants preserving cellular redox homeostasis through different enzymatic antioxidant mechanisms. In *B. asiatica*, SOD and POD activities contributed greatly to the detoxification mechanism in response to oxidative stress, while *B. macrostachya* relied only on POD activity. Moreover, both species showed great Cd tolerance and resistance, and both are therefore potential Cd-hypertolerant plants for use in phytoremediation.

## Materials and methods

### Cultivation of plants and soil treatments

This study was carried out between April 2017 and February 2019 in the greenhouses of Honghe University, Yunnan, China. Cuttings were taken from a mature *B*. *asiatica* specimen and biennial seedlings of *B*. *macrostachya* were used. Before the experiments, all individuals (about 40) were collected and cultivated in a greenhouse at Honghe University for 1 week. Twenty-five individuals of each species, having similar heights and growth vigor were then selected for use in the experiments.

Soil was collected from the base of the mature *B. asiatica* specimen and was used in each of the experiments. The organic carbon content of the soil sample was 32.85 ± 8.18 mg g^−1^, the total nitrogen was 0.34 ± 0.10 mg g^−1^, and the total phosphorous was 0.20 ± 0.02 mg g^−1^. The soil sample was slightly Cd-contaminated, and Cd content was ~ 4.60 mg kg^−1^. Soil samples were air-dried and sieved through a 2 mm mesh, then subpackaged into separate bags with 10 kg in each bag. The levels of Cd in the soil were then adjusted to 0, 25, 50, 100, and 200 mg kg^−1^ DW using Cd supplied as Cd(NO_3_)_2_·4H_2_O. All soil samples were then watered with distilled water and incubated in bags for 20 days with ~ 60% humidity^41^. Plants were then transplanted into the soil and were cultivated under normal greenhouse conditions for 90 days.

### Determination of Cd concentration

Following exposure to Cd for 90 days, the Cd concentrations in different plant tissues were investigated. Plant samples were washed clean of soil and were rinsed with deionized water. Subsequently, plant roots, stems, and leaves were separately oven-dried at 70 °C for a week. The dried tissues were weighed, ground, and sieved through a 2 mm stainless steel mesh.

Plant tissues (roots, stems, and leaves) were separately microwave digested using the Speedwave ENTRY system (Berghof, Germany) with a mixed solution of HNO_3_ + HClO_4_ (v/v) = 3:1. The Cd content was determined using Flame Atomic Absorbance Spectrometry (FAAS; TAS-990, Beijing Purkinje General Instrument Co., Ltd, China) and a standard Cd solution (GSB04-1721-2004) provided by the National Center for Reference Materials.

### Evaluation of Cd translocation potential

To evaluate the potential of the test plants to bioaccumulate Cd, the translocation factor (TF) and bioconcentration factor (BCF) indexes were further calculated following Liu et al.^29^:

$$ {\text{TF }} = \, \left[ {{\text{Cd}}} \right]_{{{\text{shoot}}}} / \, \left[ {{\text{Cd}}} \right]_{{{\text{root}}}} $$

$$ {\text{BCF }} = \, \left[ {{\text{Cd}}} \right]_{{\text{shoot or root}}} / \, \left[ {{\text{Cd}}} \right]_{{{\text{soil}}}} $$

### Evaluation of photosynthetic activity

Chlorophyll *a* fluorescence traits were recorded using a Li-6400XT with a fluorescent leaf chamber (LiCOR Inc., USA). Ten mature leaves from each treatment were selected for the measurement of chlorophyll *a* fluorescence and were marked. After being dark-adapted for 30 min, Fo and Fm were recorded. The ratio of variable to maximal fluorescence [Fv/Fm; Fv/Fm = (Fm − Fo)/ Fm], which characterizes the quantum yield of photosystem II, was determined following Figueroa et al.^31^.

The following day (9:00–11:00), chlorophyll *a* fluorescence parameters were measured using the same leaf. Before the test, all marked leaves were light-adapted with natural light for half an hour. A saturating light pulse (1200 µmol quanta m^2^ s^−1^, 1 s) was applied for closing all reaction centers. Then Fo′, Fm′, and Fs were recorded^5^.

On top of this, the effective quantum yield of PSII [ΦPSII; ΦPSII = (Fm′ − Fs)/Fm′] was calculated following Redondo-Gómez et al.^42^. The non-photochemical quenching [NPQ; NPQ = (Fm − Fm′)/Fm′], photochemical quenching [Qp; Qp = 1 − (Fs − Fo′)/(Fm′ − Fo′)], and photochemical efficiency of PSII in light (Fv′/Fm′) were calculated following Lima et al.^35^ and Ware et al*.*^43^. The quantum yield of non-regulated energy dissipation [Y(NO); Y(NO) = Fs/Fm] and quantum of regulated energy dissipation [Y(NPQ); Y(NPQ = 1 − Y(II) − Y(NO)] were further calculated according to Huang et al.^33^.

### Determination of lipid peroxidation

To evaluate the extent of lipid peroxidation of plants, the concentrations of malondialdehyde (MDA) in the leaves were measured following Sun et al.^17^. The seventh or eighth healthy mature leaves from the apex of the branches were used for analysis. About 0.5 g of fresh leaf sample was ground and dissolved in 10 ml cold trichloroacetic acid (TCA; 10%). The homogenate was then centrifuged for 20 min at 4000 rpm and 4 °C for 10 min. The liquid supernatant was taken and used for analysis. Three replicates for each Cd treatment were performed.

### Assay of antioxidant enzymes

To evaluate the antioxidant enzyme defense system in the study plants, the antioxidant metabolism was investigated. The seventh or eighth healthy mature leaves from the apex of the branches were used for analysis. About 0.2 g of fresh leaf tissues was ground and dissolved in 10.0 ml of cold phosphatic buffer solution (PBS) at PH 7.0. The homogenate was then centrifuged at 4000 rpm for 15 min. The supernatant liquid was taken and used for analysis. Superoxide dismutase (SOD), peroxidase (POD), and catalase (CAT) activities were subsequently determined following Sun et al.^17^ and Sidhu et al.^11^. Three replicates for each Cd treatment were performed. The SOD activity was determined by measuring the inhibition in the photoreduction of nitroblue tetrazolium (NBT). The POD activity was evaluated using the guaiacol oxidation assay. The CAT activity was determined by monitoring the disappearance of H_2_O_2_.

### Statistical analyses

To evaluate the performance and acclimatization of plants under different Cd stresses, the membership function value of Cd resistance in each *Buddleja* species was also calculated using the modified equations following Liu et al.^39^ as follows:

1 $$ {u\left( {X_{j} } \right) = \frac{{\left( {X_{j} - X_{{{\text{min}}}} } \right)}}{{\left( {X_{{{\text{max}}}} - X_{{{\text{min}}}} } \right)}}},\;\;{\text{ j }} = { 1},{2}, \ldots ,{\text{n}} $$

2 $$ {{W_{j} } = \left| {P_{j} } \right|/\sum\limits_{{j = {1}}}^{n} {\left| {P_{j} } \right|} },\;\;{\text{j }} = { 1},{2}, \ldots ,{\text{n}} $$

3 $$ {D = \sum\limits_{{j = {1}}}^{n} {\left[ {u(X_{j} ) \times W_{j} } \right]} },\;\;{\text{j }} = { 1},{2}, \ldots ,{\text{n}} $$

where *u*(*X*_*j*_) is the membership function value for adaptability of the trait (j); *X*_*j*_, *X*_*min*_, and *X*_*max*_ are the means, minimum and maximum values of the trait (j), respectively; *P*_*j*_ is the contribution rate of the trait (j) based on principal component analysis; *W*_*j*_ is rate of the contribution of the trait (j) in all studied traits, and D is the comprehensive membership function value for the adaptability of the species.

One-way analysis of variance (ANOVA) was performed using PAST version 2.0 to reveal any differences between species or different Cd treatments. Fisher’s least significant difference (LSD) tests were performed using PAST version 2.0^44^ to analyze statistically significant differences among different samples at the level of 0.05 and 0.01. A significant difference was considered at two different levels (*p* < 0.05 or < 0.01). Unless indicated, data presented represent the mean value plus or minus the standard error (± SE). To reduce the heterogeneity of variances, the data were log_10_-transformed, if necessary. All bar charts were created by using ORIGIN (Version 2019b, OriginLab Corporation, USA) and then adjusted and assembled using Adobe Illustrator CS4 software (Adobe Systems, San Jose, CA) as shown in Figs. 1 and 2.

## References

[1] Benavides MP, Gallego SM, Tomaro ML (2005). Cadmium toxicity in plants. Braz. J. Plant Physiol..

[2] Sharma SS, Dietz KJ (2009). The relationship between metal toxicity and cellular redox imbalance. Trends Plant Sci..

[3] Gill SS, Khan NA, Tuteja N (2012). Cadmium at high dose perturbs growth, photosynthesis and nitrogen metabolism while at low dose it up regulates sulfur assimilation and antioxidant machinery in garden cress (*Lepidium sativum* L.). Plant Sci..

[4] Moustaka J, Tanou G, Adamakis ID, Eleftheriou EP, Moustakas M (2015). Leaf age dependent photoprotective and antioxidative mechanisms to paraquat-induced oxidative stress in *Arabidopsis thaliana*. Int. J. Mol. Sci..

[5] Ruley AT, Sharma NC, Sahi SV, Singh SR, Sajwan KS (2006). Effects of lead and chelators on growth, photosynthetic activity and Pb uptake in *Sesbania drummondii* grown in soil. Environ. Pollut..

[6] Mobin M, Khan NA (2007). Photosynthetic activity, pigment composition and antioxidative response of two mustard (*Brassica juncea*) cultivars differing in photosynthetic capacity subjected to cadmium stress. J. Plant Physiol..

[7] Najeeb U (2011). Insights into cadmium induced physiological and ultra-structural disorders in *Juncus effusus* L. and its remediation through exogenous citric acid. J. Hazard Mater..

[8] Li X, Zhao M, Guo L, Huang L (2012). Effect of cadmium on photosynthetic pigments, lipid peroxidation, antioxidants, and artemisinin in hydroponically grown *Artemisia annua*. J. Environ. Sci..

[9] Anjum SA (2015). Morphophysiological growth and yield responses of two contrasting maize cultivars to cadmium exposure. Clean Soil Air Water.

[10] Khan MIR, Nazir F, Asgher M, Per TS, Khan NA (2015). Selenium and sulfur influence ethylene formation and alleviate cadmium induced oxidative stress by improving proline and glutathione production in wheat. J. Plant Physiol..

[11] Sidhu GPS, Singh HP, Batish DR, Kohli RK (2017). Tolerance and hyperaccumulation of cadmium by a wild, unpalatable herb *Coronopus didymus* (L.) Sm. (Brassicaceae). Ecotoxicol. Environ. Saf..

[12] Sharma P, Dubey RS, Khan NA, Samiullah S (2006). Cadmium uptake and its toxicity in higher plants. Cadmium Toxicity and Tolerance in Plants.

[13] Anjum NA, Khan NA, Sofo A, Baier M, Kizek R (2016). Editorial: Redox homeostasis managers in plants under environmental stresses. Front. Environ. Sci..

[14] Semane B (2010). Leaf proteome responses of *Arabidopsis thaliana* exposed to mild cadmium stress. J. Plant Physiol..

[15] Asgher M, Khan MIR, Anjum NA, Khan NA (2015). Minimizing toxicity of cadmium in plants—Role of plant growth regulators. Protoplasma.

[16] Hegedüs A, Erdei S, Horváth G (2001). Comparative studies of H_2_O_2_ detoxifying enzymes in green and greening barley seedlings under cadmium stress. Plant Sci..

[17] Sun Y, Zhou Q, Wang L, Liu W (2009). Cadmium tolerance and accumulation characteristics of *Bidens pilosa* L. as a potential Cd-hyperaccumulator. J. Hazard Mater..

[18] Anjum NA (2016). Catalase and ascorbate peroxidase—Representative H_2_O_2_-detoxifying heme enzymes in plants. Environ. Sci. Pollut. Res..

[19] Murtaza B (2019). A multivariate analysis of physiological and antioxidant responses and health hazards of wheat under cadmium and lead stress. Environ. Sci. Pollut. Res..

[20] Slama I, Abdelly C, Bouchereau A, Flowers T, Savouré A (2015). Diversity, distribution and roles of osmoprotective compounds accumulated in halophytes under abiotic stress. Ann. Bot..

[21] Smokvarska M, Francis C, Platre M, Martinière A (2020). A Plasma membrane nanodomain ensures signal specificity during osmotic signaling in plants. Curr. Biol..

[22] Kalaji HM (2016). Chlorophyll a fluorescence as a tool to monitor physiological status of plants under abiotic stress conditions. Acta Physiol. Plant.

[23] Wu M (2017). Physiological and biochemical mechanisms preventing Cd toxicity in the new hyperaccumulator *Abelmoschus manihot*. J. Plant Growth Regul..

[24] Guidi L, Landi M, Penella C, Calatayud A (2016). Application of modulated chlorophyll fluorescence and modulated chlorophyll fluorescence imaging in studying environmental stresses effect. Annali di Botanica.

[25] Norman, E. M. Buddlejaceae. Flora neotropica monograph, vol. 81, 1–190 (The New York Botanical Garden, Bronx, 2000).

[26] Waranusantigul P, Kruatrachue M, Pokethitiyook P, Auesukaree C (2008). Evaluation of Pb phytoremediation potential in *Buddleja asiatica* and *B. paniculata*. Water Air Soil Pollut..

[27] Qi C (2011). Distribution and accumulation of antimony in plants in the super-large Sb deposit areas, China. Microchem. J..

[28] Tian SK (2017). Uptake, sequestration and tolerance of cadmium at cellular levels in the hyperaccumulator plant species *Sedum alfredii*. J. Exp. Bot..

[29] Liu Z (2009). Accumulation and tolerance characteristics of cadmium in a potential hyperaccumulator–*Lonicera japonica* Thunb. J. Hazard Mater..

[30] Figueroa ME, Fernández-Baco L, Luque T, Davy AJ (1997). Chlorophyll fluorescence, stress and survival in populations of *Mediterranean grassland* species. J. Veg. Sci..

[31] Björkman O, Demmig B (1987). Photon yield of O_2_ evolution and chlorophyll fluorescence characteristics at 77 K among vascular plants of diverse origins. Planta.

[32] Sheng M (2008). Influence of arbuscular mycorrhizae on photosynthesis and water status of maize plants under salt stress. Mycorrhiza.

[33] Huang W, Zhang SB, Cao KF (2010). Stimulation of cyclic electron flow during recovery after chilling-induced photoinhibition of PSII. Plant Cell Physiol..

[34] Zaiyou J, Xiu-ren Z, Jing T (2020). Photosynthetic and chlorophyll fluorescence characteristics of *Isodon rubescens* (Hemsley) H. Hara. Sci. Rep..

[35] Lima CS (2018). Antioxidant protection and PSII regulation mitigate photo-oxidative stress induced by drought followed by high light in cashew plants. Environ. Exp. Bot..

[36] Tsikas D (2017). Assessment of lipid peroxidation by measuring malondialdehyde (MDA) and relatives in biological samples: Analytical and biological challenges. Anal. Biochem..

[37] Pandhair V, Sekhon BS (2006). Reactive oxygen species and antioxidants in plants: An overview. J. Plant Biochem. Biotechnol..

[38] Guo J (2019). Cadmium stress increases antioxidant enzyme activities and decreases endogenous hormone concentrations more in Cd-tolerant than Cd-sensitive wheat varieties. Ecotoxicol. Environ. Saf..

[39] Liu N (2017). Evaluation of mercury resistance and accumulation characteristics in wheat using a modified membership function. Ecol. Indic..

[40] Yan C (2020). Screening diverse soybean genotypes for drought tolerance by membership function value based on multiple traits and drought-tolerant coefficient of yield. BMC Plant Biol..

[41] Huang H (2011). The phytoremediation potential of bioenergy crop *Ricinus communis* for DDTs and cadmium co-contaminated soil. Bioresour. Technol..

[42] Redondo-Gómez S (2006). Growth and photosynthetic responses to salinity in an extreme halophyte, *Sarcocornia fruticosa*. Physiol. Plantarum.

[43] Ware MA, Belgio E, Ruban AV (2015). Photoprotective capacity of non-photochemical quenching in plants acclimated to different light intensities. Photosynth. Res..

[44] Hammer Ø, Harper DAT (2006). Paleontological Data Analysis.

